# EMAGINE–Study protocol of a randomized controlled trial for determining the efficacy of a frequency tuned electromagnetic field treatment in facilitating recovery within the subacute phase following ischemic stroke

**DOI:** 10.3389/fneur.2023.1148074

**Published:** 2023-05-05

**Authors:** Jeffrey L. Saver, Pamela W. Duncan, Joel Stein, Steven C. Cramer, Janice J. Eng, Assaf Lifshitz, Arielle Hochberg, Natan M. Bornstein

**Affiliations:** ^1^Department of Neurology, University of California, Los Angeles, Los Angeles, CA, United States; ^2^School of Medicine, Wake Forest University, Winston-Salem, NC, United States; ^3^Weill Cornell Medicine, Cornell University, White Plains, NY, United States; ^4^California Rehabilitation Institute, Los Angeles, CA, United States; ^5^Department of Physical Therapy, University of British Columbia, Vancouver, BC, Canada; ^6^BrainQ Technologies Ltd., Jerusalem, Israel; ^7^Brain Division, Shaare Zedek Medical Center, Jerusalem, Israel

**Keywords:** subacute stroke, ELF-EMF, stroke recovery, neurostimulation, non-invasive, electromagnetic field, motor recovery, upper extremity motor impairment

## Abstract

Stroke is a leading cause of disability with limited effective interventions that improve recovery in the subacute phase. This protocol aims to evaluate the safety and efficacy of a non-invasive, extremely low-frequency, low-intensity, frequency-tuned electromagnetic field treatment [Electromagnetic Network Targeting Field (ENTF) therapy] in reducing disability and promoting recovery in people with subacute ischemic stroke (IS) with moderate-severe disability and upper extremity (UE) motor impairment. Following a sample-size adaptive design with a single interim analysis, at least 150 and up to 344 participants will be recruited to detect a 0.5-point (with a minimum of 0.33 points) difference on the modified Rankin Scale (mRS) between groups with 80% power at a 5% significance level. This ElectroMAGnetic field Ischemic stroke–Novel subacutE treatment (EMAGINE) trial is a multicenter, double-blind, randomized, sham-controlled, parallel two-arm study to be conducted at approximately 20 United States sites, and enroll participants with subacute IS and moderate-severe disability with UE motor impairment. Participants will be assigned to active (ENTF) or sham treatment, initiated 4–21 days after stroke onset. The intervention, applied to the central nervous system, is designed for suitability in multiple clinical settings and at home. Primary endpoint is change in mRS score from baseline to 90 days post-stroke. Secondary endpoints: change from baseline to 90 days post-stroke on the Fugl-Meyer Assessment - UE (lead secondary endpoint), Box and Block Test, 10-Meter Walk, and others, to be analyzed in a hierarchical manner. EMAGINE will evaluate whether ENTF therapy is safe and effective at reducing disability following subacute IS.

**Trial registration:**

www.ClinicalTrials.gov, NCT05044507 (14 September 2021).

## Introduction

Stroke is a leading cause of long-term disability, especially as mortality rates are declining (1, 2). Given the aging population and increased stroke risk, annual stroke-related costs in the United States (US) are projected to reach $240.67 billion by 2030 (2). Reperfusion interventions are beneficial but invasive, are only available in the most acute stages, require skilled personnel, and are limited to eligible patients; 73% have a disabled or fatal outcome by 90 days (3).

Beyond the acute phase (1–7 days) (4) and into the early subacute phase (< 3 months) (4), stroke rehabilitation focuses on physical, occupational and speech therapies (PT, OT, SLP). However, standard of care (SOC) varies across facilities, and only a fraction of patients completely recover (5, 6). Moreover, the treatment pathway is fragmented, with patients treated in a variety of inpatient and outpatient clinical settings as well as at home (6). Preclinical (7, 8) and clinical trials (9, 10) indicate heightened plasticity in the post-stroke brain that declines in the first few weeks, highlighting the importance of early intervention. There is an urgent need for effective and accessible early subacute therapy that is suitable across multiple settings.

Following injury, neuronal network connectivity is disrupted, with aberrant oscillatory patterns on electroencephalography (EEG) (11). As network dynamics are sensitive to external electromagnetic fields at specific frequencies (12–14), the proposed mechanism of action of the experimental treatment involves exposing impaired neuronal networks to oscillating fields similar to those of a healthy central nervous system to induce neuroprotective cellular mechanisms and promote network reorganization (13, 15–17). Prominent frequencies of these oscillations were identified using EEG recordings of healthy and impaired populations and translated into a non-invasive, extremely low-frequency, low-intensity, frequency-tuned electromagnetic field treatment [Electromagnetic Network Targeting Field (ENTF) therapy].

Preclinical results suggest that ENTF therapy post-stroke impacts cellular mechanisms and network reorganization (18, 19). In a rodent stroke model, oscillating extremely low-frequency, low-intensity electromagnetic fields (ELF-EMF) stimulation (exposure to sham field, 3.93 Hz or 15.72 Hz, every second day, for 4 weeks) was associated in treated animals with decreased edema, increased white matter integrity, evidence of neural regeneration, and improved sensorimotor function on the modified Neurological Severity Score and forelimb placement test (18). Overall, data suggest that such treatment targets functional neural networks, promotes neural plasticity and modulates the secondary injury cascade, thus aiding clinical recovery.

A pilot randomized controlled trial (*n* = 21) found that ENTF therapy delivered in the early subacute phase (3–15 days post-ischemic stroke; 21 days if unstable) increased upper extremity (UE) motor function across multiple metrics and reduced disability (20). This was observed by a greater improvement with ENTF compared to sham stimulation on the trial primary outcome of the Fugl-Meyer Assessment–Upper Extremity (FMA-UE): from baseline to week 4 (23.2 ± 14.1 vs. 9.6 ± 9.0, *p* = 0.007); baseline to week 8 (31.5 ±10.7 vs. 23.1 ± 14). Similar favorable effects at week 8 were observed for other UE assessments, including the Action Research Arm Test (Pinch, 13.4 ± 5.6 vs. 5.3 ± 6.5, *p* = 0.008) and Box and Blocks Test (affected hand, 22.5 ± 12.4 vs. 8.5 ± 8.6, *p* < 0.0001). Reduction of global disability was assessed by the modified Rankin Scale (mRS) (20), a global outcome measure scored from 0 (no symptoms) to 6 (death). At baseline, participants were moderate-severely disabled (mRS 3-4) and by day 70 post-stroke, the ENTF therapy group improved by a mean 2.5 (±0.66) points relative to 1.3 (±0.46) points for the sham group. As a comparison, in a novel analysis of data from large trials (detailed in Supplementary File 1), moderate-severely disabled patients, with SOC treatment, improved by a mean of ~1 point by day 90 post-stroke.

The ElectroMAGnetic field Ischemic stroke–Novel subacutE treatment (EMAGINE) trial investigates the impact of ENTF therapy, on disability and functional recovery, in conjunction with SOC; ENTF treatment is introduced within 3 weeks post-stroke, a period in which the post-stroke brain has heightened plasticity potential (7–10). ENTF therapy involves non-invasive stimulation that is suitable and easy to use in multiple settings, including at home. To date, there have been no serious adverse events; EMAGINE is being conducted as a non-significant risk device study.

The primary objective of EMAGINE is to determine the efficacy and safety of ENTF therapy in reducing disability in the subacute phase post-stroke. The hypothesis for efficacy is that mean improvement in the primary outcome, mRS score, from baseline to 90 days post-stroke will be significantly greater in participants allocated to active stimulation (ENTF group) than in participants allocated to sham stimulation (sham) group.

## Materials and methods

### Formal study reporting methods

This methods manuscript adheres to the methodology of the Standard protocol items: recommendations for interventional trials (SPIRIT) statement and checklist. Additionally, the description of the study intervention adheres to the methodology of the Template for Intervention Description and Replication statement and checklist (TIDieR). The primary results paper will adhere to the methodology of the Consolidated Standards of Reporting Trials (CONSORT) statement and checklist.

### Study design

EMAGINE is a multicenter, double-blind, randomized, sham-controlled, parallel, two-arm study following a sample-size-adaptive design with a single planned interim analysis. The study will be conducted at approximately 20 US-based acute care and inpatient rehabilitation facilities (IRFs) with enrollment from any site not exceeding 20% of the total sample size. Informed consent will be obtained 3–21 days post-stroke, and participation will continue up to 180 (±15) days post-stroke.

**Figure 1:**
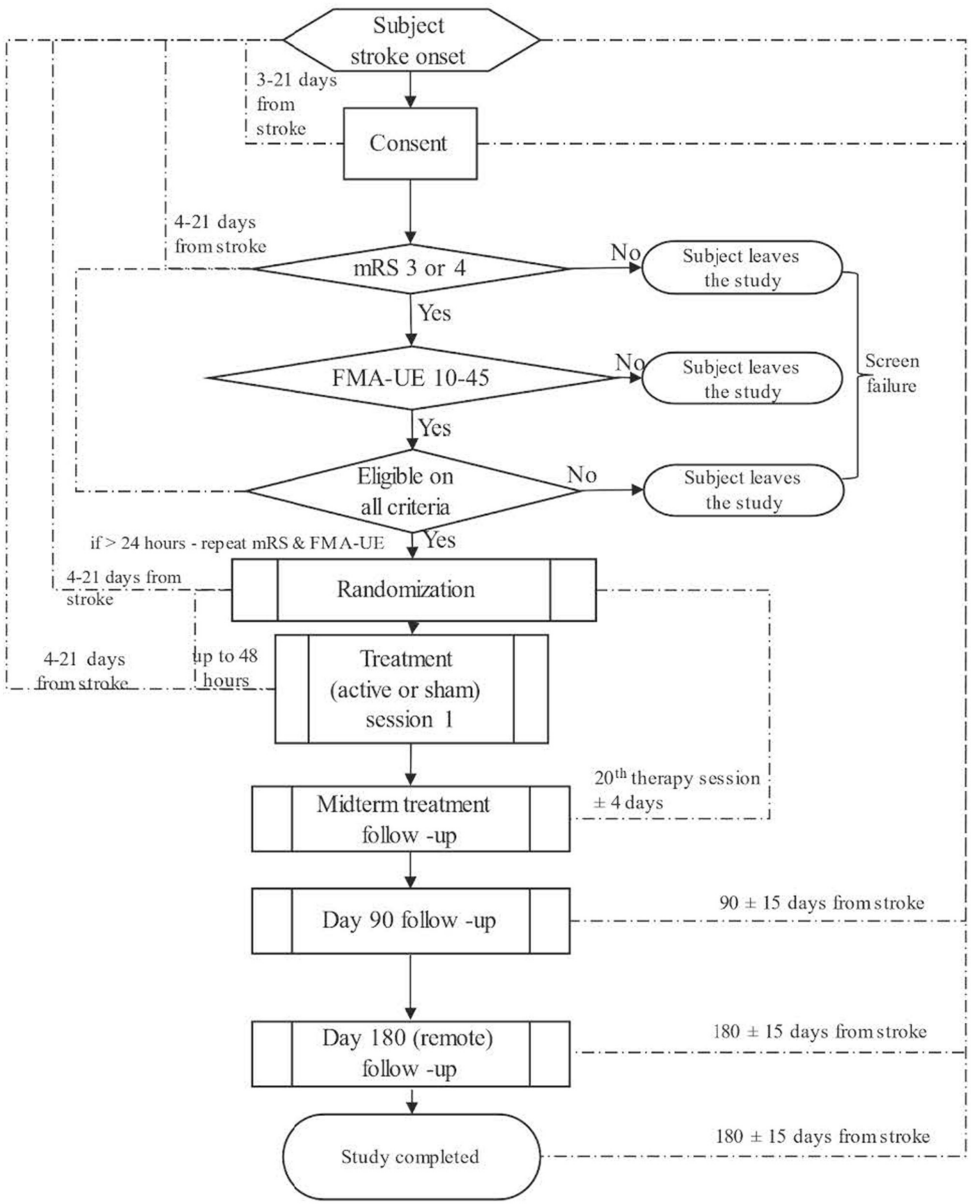
Trial design.

Participants will undergo initial screening and detailed baseline assessment, followed by 45 treatment sessions over 9 weeks. Follow-up assessments will be at the 20th treatment session (±4 days), 90 (±15) days post-stroke, and 180 (±15) days post-stroke (long-term outcome; global disability and quality of life measures) (Figure 1).

### Patient population–Inclusion and exclusion

Prospective participants 3–21 days post-stroke will be consented to enter the study screening phase and at 4–21 days post-stroke, participants fully meeting entry criteria will proceed to the randomization phase and be allocated to a study treatment arm. This ensures that treatment will begin soon after stroke onset, when the brain has heightened plasticity potential (7–10). Key inclusion criteria: mRS score of 3 or 4; Fugl-Meyer Assessment (FMA) score of 10–45 (of 66) for impaired UE (this range was informed by the results of the pilot study, which demonstrated improvements after 4 and 8 weeks, and allows for further improvement on the scale); 22–85 years of age; diagnosed with an ischemic stroke, confirmed by CT or MRI; 4–21 days post-stroke onset; pre-stroke mRS of 0 or 1; and availability of a relative or caregiver to assist with treatment. Key exclusion criteria: implanted active electronic or passive MR-incompatible device; ischemic or hemorrhagic stroke within 2 weeks prior to index stroke (participants with prior strokes that occurred more than 2 weeks prior to the index will be allowed for inclusion); pre-existing neurological condition or physical limitation that would significantly interfere with participation; active epilepsy or taking anti-epileptic medication or seizure in last 5 years; and unstable serious illness/condition or life expectancy of < 12 months. Research involving Human Participants, approval of the protocol and the informed consent form was obtained from a central Institutional Review Board (IRB) before any participant was consented/enrolled. Informed consent will be obtained from all participants included in the study. The detailed inclusion and exclusion criteria can be found in Supplementary Table 1, ethics in Supplementary File 2 and recruitment strategies in Supplementary File 3.

### Randomization

Participants will be equally allocated (1:1 ratio) based on a permuted block randomization scheme, stratified by site, age (22–69, 70–85 years) and baseline mRS (3 or 4) to either the active (ENTF) or sham stimulation group. An authorized, trained, unblinded individual at the site will enter group assignment into the device based on a pre-programmed randomization algorithm embedded in an electronic data capture system (Syncrony). Participants, caregivers, outcome assessors, site investigators (aside from the one unblinded individual responsible for group assignment), and the sponsor will be unaware of group assignment. The device produces no noticeable sound, light or sensation in connection with stimulation, facilitating blinding. Blinding methods will incorporate appropriate security measures and access control. In an emergency, investigators may determine that a participant be unblinded.

### Intervention

The investigational medical device (BQ 2.0; BrainQ Technologies Ltd., Jerusalem, Israel) delivers a non-invasive, extremely low-frequency (1–100 Hz) and intensity ( ≤ 1 Gauss), frequency-tuned electromagnetic field. The device is portable, wearable and designed for use in multiple settings (e.g., clinic, home); it includes embedded magnetic coils, a mobile device with a dedicated app that guides the treatment session, and a single-use adhesive electromagnetic sensor placed on the forehead to monitor treatment (the data from the sensor will not be reviewed or analyzed for the duration of the study in order to maintain blinding). The device can be used by a professional operator or lay person (i.e., caregiver). The device does not require installation and it comes with all the necessary equipment and instructions for use. A video demonstrating the application of the device and general overview of the treatment session can be found in Supplementary Video 1. The device technology utilized explanatory machine learning and brain-computer interface-based tools to identify relevant spectral patterns based on a database of EEG recorded during functional motor tasks. These spectral patterns were then translated into ENTF therapy that applies similar patterns directly to the participant's head and spine.

The first treatment session will be within 48 h from randomization and 4–21 days from stroke onset. There will be 45 treatment sessions over 9 weeks (5 per week). Each session will last up to 60 min, with ENTF or sham field applied for 40 min together with an evidence-based, functional, repetitive and graded PT/OT regimen (21) guided by an app. The PT/OT will include approximately 30 min dedicated to UE exercises and 30 min to lower extremity (20 min without the device). This is consistent with Class 1 Level A recommendation for people with stroke to perform functional, task-specific, graded and repetitive tasks, and participate in home-based rehabilitation (6). Adherence to treatment is captured in the electronic data capture system and by data logs collected via the app.

A trained site study team member (trained by sponsor personnel) will be responsible for training the participant's caregiver to operate sessions independently, whether in the clinic or at home. Sessions will be supervised, in person or remotely, until the caregiver is deemed capable of operating sessions independently with periodic study team oversight.

### Concomitant therapy

All sites will provide concomitant SOC medical and rehabilitation therapies to prevent recurrent stroke and maximize recovery according to US national guidelines (6).

### Clinical outcomes

The primary efficacy, secondary efficacy and exploratory endpoints are listed below, with a description of the clinical relevance of the main endpoints.

#### Primary efficacy endpoint

EMAGINE's primary outcome measure is the modified Rankin Scale (mRS; global disability) change from baseline (4–21 days post-stroke) to 90 days post-stroke. The mRS is a broad scale with 7 levels from normal through increasing disability to death (scored 0–6). The modified Rankin Scale is the most frequently used outcome measure in stroke trials (22). The mRS measures the degree of disability or dependence in the daily activities of people who have suffered a stroke or other causes of neurological disability (23). In wide use, the mRS shows strong construct validity, with strong correlations with prognostic indicators such as stroke type, lesion size, etc., in addition to convergent validity between mRS and other related disability scales (24–26).

A known limitation of the original mRS is score assignment based on poorly specified, non-operationalized distinctions between levels, leading to poor reproducibility of the score by various examiners (27, 28). Inter-rater reliability of the original, holistic mRS scoring process is especially reduced in multicenter studies employing many raters (28). Initiatives to improve the use of the mRS have included: (1) training and certification of examiners, (2) structured interviews and questionnaires, and (3) centralized review of videotape assessments (29). However, substantial interobserver variability in mRS assessment persists even following the certification of assessors or the use of structured interviews (26, 29).

Given these limitations, the Rankin Focused Assessment (RFA) was developed with clear operationalized criteria for evaluating and determining disability levels; the RFA has high inter-rater reliability when used in a randomized control trial setting (30).

To increase outcome comparability and minimize bias, the mRS assessments of global disability in the EMAGINE trial are obtained using the formal, algorithmic Rankin Focused Assessment-Ambulatory (RFA-A) method by RFA-A-certified raters (30, 31). A panel of independent blinded experts will remotely and centrally assess the mRS (using the RFA), as well as a set of additional questionnaire-based secondary and exploratory outcomes - Stroke Impact Scale - Hand Domain (SIS-Hand), Stroke Impact Scale-16 (SIS-16), 5-level EuroQol-5 Dimension (EQ-5D-5L), 8-Item Patient Health Questionnaire (PHQ-8), and Academic Medical Center (AMC) Linear Disability Score (ALDS).

Remote centralized assessment of the mRS via telehealth tools has been established in other trials (32–34). Consistent remote centralized assessment via telehealth will minimize inconsistency among assessors across timepoints, enhance level of expertise, and minimize interrater variability across the trial. An additional advantage of using a centralized rating panel is that it reinforces the maintenance of blinding in outcome assessment.

#### Secondary efficacy endpoints

Secondary efficacy endpoints will be analyzed in the following hierarchical order:

- Fugl-Meyer Assessment for Upper Extremity (FMA-UE; upper limb function) – change from baseline (4–21 days post-stroke) to 90 days post-stroke. The Fugl-Meyer Assessment–Upper Extremity (FMA-UE) is a performance-based impairment index designed to assess motor functioning, balance, sensation, and joint function in patients with post-stroke hemiparesis (35). FMA-UE is frequently applied clinically and in research to determine disease severity and describe motor recovery in order to plan and assess rehabilitation (36). The FMA-UE is one of the most common instruments in rehabilitation and has established validity, reliability, and sensitivity to treatment-related change (25, 37–39)
- Box and Block Test (BBT; arm motor function)–change from baseline (4–21 days post-stroke) to 90 days post-stroke. The BBT (40) measures unilateral gross manual dexterity, and has shown reliability and validity in multiple studies of post-stroke patients (41).
- 10-Meter Walk Test (10MWT; gait speed)–change from baseline (4–21 days post-stroke) to 90 days post-stroke.^1^
- Stroke Impact Scale - Hand Domain (SIS-Hand; patient-reported hand function)–change from baseline (4–21 days post-stroke) to 90 days post-stroke.
- Stroke Impact Scale - 16 (SIS-16; patient-reported physical and functional limitation) –change from baseline (4-21 days post-stroke) to 90 days post-stroke.
- 5-level EuroQol-5 Dimension (EQ-5D-5L; health-related quality of life) at 90 days.

#### Safety endpoints

- Serious procedure- or device-related adverse events (AE).
- Device deficiencies to detect operational reliability.

#### Exploratory endpoints

- Montreal Cognitive Assessment (MoCA; global cognitive function)–at 90 days post-stroke.
- Patient Health Questionnaire-8 (PHQ-8; depression)–at 90 days post-stroke.
- Academic Medical Center Linear Disability Scale (ALDS; granular level of disability) at 90 days post-stroke.
- modified Rankin Scale (mRS; global disability)–change from baseline to 180 days post-stroke.
- Stroke Impact Scale - Hand Domain (SIS-Hand; patient-reported hand function)–change from baseline to 180 days post-stroke.
- 5-level EuroQol-5 Dimension (EQ-5D-5L; health-related quality of life) at 180 days post-stroke.
- Formal cost-effectiveness analysis over a lifetime horizon from the perspective of the United States healthcare system. An expert in cost-effectiveness analysis will provide the planned analysis for inclusion in the final statistical analysis plan prior to unblinding.
- Relationship between adherence to treatment and the clinical outcomes as measured by the adhesive electromagnetic sensor. Treatment adherence impact will be explored using correlation analysis, odds ratios, and cluster analysis (42).

All assessments are performed by blinded assessors. To reduce inter-rater variability and enhance data integrity, in all participants nationally, the primary endpoint, as well as a set of additional questionnaire-based secondary and exploratory outcomes (SIS-16, SIS hand domain, EQ-5D-5L, PHQ-8, ALDS), will be assessed via telehealth tools by a centralized expert rater. The small panel of central expert raters will be highly experienced in outcome assessment and specifically certified in mRS administration. The remaining endpoints, which require physical presence (e.g., FMA-UE, BBT, 10MWT) or access to records (e.g., cost-effectiveness endpoint) are assessed or collected by blinded site clinical staff who underwent training and certification.

### Data monitoring body

An independent Data and Safety Monitoring Board (DSMB) will be established to oversee trial safety and efficacy. The DSMB will provide recommendations regarding recruitment, enrollment, AEs, modifying, or stopping the trial based on data review. The board will meet at least semiannually, either in person or by teleconference.

### Sample size estimate

Sample size was calculated to detect a 0.5-point difference between groups with 80% power at a 5% two-tailed level of significance, assuming a standard deviation (SD) of 1 point and 1:1 allocation ratio. The calculated sample size (PROC POWER in SAS V9.4) is 128 total participants, 64 participants per arm. Allowing for a 15% dropout rate, 150 participants should be randomized.

Estimates for mean and SD of changes from baseline to Day 90 are taken from literature and pilot data (20, 43). In the pilot study, mean change in mRS score from baseline to Day 70 was 2.5 (±0.66) for ENTF vs. 1.3 (±0.46) for sham. A 0.5-point difference on the mRS is roughly 40% of the difference in the pilot study (20) and above the clinically meaningful difference (22). One interim analysis is planned after 61% of evaluable participants complete the Day 90 visit, with rules for continuation to the original sample size, reassessment (allowance for up to 344 participants), or stopping due to futility (44, 45). Interim analysis will be performed by an independent unblinded statistician, and the DSMB will inform sponsor and investigators of its recommendation.

### Statistical analyses

Study data will be summarized with descriptive statistics presenting: count and percent for categorical and discrete data, mean, standard deviation, minimum, and maximum for normally distributed continuous variables, and median, interquartile range, minimum and maximum for non-normally distributed continuous variables. Demographic and other baseline data will be compared between the groups. For comparison of continuous variables, two-sample *t*-test or Wilcoxon rank sum test will be used. For comparison of proportions (categorical variables), chi-squared test or Fisher's exact test will be used.

The primary endpoint, mRS change from baseline to Day 90, will be compared between treatment groups using repeated measures analysis of covariance (fixed effects: treatment group, visit (midterm and Day 90 follow-up visit), treatment group by visit interaction with baseline mRS and age entered as covariates, random effect: site). Shift analysis of the primary endpoint will be performed as a sensitivity analysis. A hierarchical approach will be adopted for primary and secondary endpoints to control type I error due to multiple endpoint testing, such that the primary endpoint will be analyzed first and, only if the null hypothesis is rejected at a significance level of ≤ 5%, will secondary endpoints be tested.

Overall significance level will be 5% using two-tailed tests. Nominal *p*-values will be reported for each endpoint even if, due to the hierarchy, the result is not considered statistically significant.

The primary safety variable, the cumulative incidence (and 95% confidence interval) of AEs reported throughout the study in each of the study groups, will be presented in tabular format and will include incidence tables by severity and relationship to study device and/or procedure. Cumulative incidence will be summarized based on the percent of participants with one or more events with the associated confidence interval. Additionally, incidence will be summarized based on the total number of events allowing for multiple events per participant (without a confidence interval). AE rates will be compared between the study groups with a Fisher's exact test.

The following analysis sets are defined for the EMAGINE study: Intent-to-treat (ITT) including all participants randomized, Modified ITT (mITT) including all participants from the ITT set for whom treatment was initiated, Per-protocol (PP) including all participants from the mITT analysis who have no clinically significant protocol deviations and were treated for a minimum of 20 completed sessions with one post-baseline data point, and Safety analyses set (SAF) including all participants who initiated treatment. The mITT analysis will serve as the principal analysis set for efficacy assessments and SAF as the principal analysis set for the analysis of safety. Further details and definitions can be found in Supplementary File 4. Further details regarding how discontinuation will be handled can be found in Supplementary File 5.

## Discussion

Despite effective SOC acute interventions and established benefits of post-stroke rehabilitation programs, many patients have significant residual disability. Long-term disability is associated with economic burden, which is projected to increase with the aging population. Annual long-term post-hospitalization costs for patients with mRS 4 are estimated at $43,755 compared to $10,883 for mRS 1 (46). EMAGINE aims to leverage neuroplasticity in the early post-stroke period to promote recovery. ENTF therapy is initiated 4-21 days post-stroke, consisting of five weekly 60-min sessions over 9 weeks. ENTF is non-invasive, complements SOC and is suitable for clinic and home administration, ensuring continuity of care. If effective in reducing disability, ENTF therapy may yield substantial gains in quality-adjusted life-years and significant cost savings.

EMAGINE is designed to assess the safety and efficacy of ENTF therapy during the subacute phase post-ischemic stroke. Given high stroke prevalence and limited treatment options beyond the acute phase, EMAGINE results may indicate the viability of a post-stroke treatment that non-invasively targets and rehabilitates compromised brain networks to reduce disability and improve quality of life. If effective, ENTF may provide accessible and scalable treatment that follows the patient from clinic to home, unifying a fragmented care pathway.

## Ethics statement

Written informed consent was obtained from the individual(s) for the publication of any identifiable images or data included in this article.

## Author contributions

JLS, PWD, and JS are coordinating principal investigators. JLS, PWD, JS, SCC, JJE, AL, AH, and NB were involved in study conception and design. AH led manuscript preparation. This manuscript was submitted by the authors on the behalf of the EMAGINE Investigators. The full list of EMAGINE Investigators and coordinators can be found in Supplementary File 6. All authors read and approved the final manuscript.

## Footnotes

^1^Participants who aren't able to walk are scored 300 seconds automatically.
