# Supplementary material for: EMAGINE–Study protocol of a randomized controlled trial for determining the efficacy of a frequency tuned electromagnetic field treatment in facilitating recovery within the subacute phase following ischemic stroke
Source: Front Neurol. 2023 May 5;14:1148074. doi: 10.3389/fneur.2023.1148074 (PMC10196621; doi:10.3389/fneur.2023.1148074)
Supplement: Supplementary file 1 [file Table_1.docx]

**Table S1 Detailed Inclusion and Exclusion Criteria**

| Inclusion criteria |
| --- |
| 1. mRS score of 3 or 4. 2. FMA-UE score between 10-45 (inclusive) for impaired limb. 3. Age 22 to 85 years (inclusive). 4. Diagnosed with an ischemic stroke, confirmed by CT or MRI imaging. 5. Four to 21 days from stroke onset (or last known well). 6. Pre-stroke mRS of 0 or 1. 7. Able to sit with the investigational device for 40 consecutive minutes, in the opinion of the investigator or designee. 8. Can follow a 3-step command, such as “take the paper, fold it in half, and return it to me.” 9. Willingness to participate in occupational/physical therapy activities during study intervention sessions. 10. Availability of a relative or other caregiver able to assist during PT/OT treatment, and to operate an application installed on a mobile device, including a video call. 11. If female, not pregnant (as confirmed by a urine or a blood test, or as determined by an official medical document) or breastfeeding and with no ability to become pregnant, or on an acceptable method of contraception during the study. 12. Informed consent signed by participant (if competent) or legally authorized representative. |
| Exclusion criteria |
| 1. Severe neglect impairment (NIHSS item 11, score = 2) or neglect that is severe enough to interfere with reasonable performance of study procedures. 2. Implanted active electronic or passive MR-incompatible devices. 3. Previous ischemic or hemorrhagic stroke within 2 weeks before the index stroke. 4. Pre-existing neurological condition (e.g., Alzheimer’s disease, Parkinson’s disease, multiple sclerosis, traumatic brain injury, spinal cord injury) or physical limitation that would interfere significantly with the participant’s participation in the study and/or confound neurological or functional evaluation. 5. Active epilepsy or currently taking anti-epileptic medication (indicated for the treatment of a seizure disorder), or seizure in the last 5 years. 6. Significant visual disturbances that cannot be corrected and that would interfere significantly with the participant’s participation in the study and/or confound neurological or functional evaluation. 7. Unstable serious illness/condition (e.g., active cancer, severe heart failure, active psychiatric condition) or life expectancy of less than 12 months. 8. A known severe allergic reaction to acrylic-based adhesives. 9. Ongoing alcohol abuse and/or illicit drug use. 10. Participation in another trial that would conflict with the current study or clinical endpoint interference may occur. 11. Employee of the sponsor. 12. Prisoner. |
