# Supplementary material for: EMAGINE–Study protocol of a randomized controlled trial for determining the efficacy of a frequency tuned electromagnetic field treatment in facilitating recovery within the subacute phase following ischemic stroke
Source: Front Neurol. 2023 May 5;14:1148074. doi: 10.3389/fneur.2023.1148074 (PMC10196621; doi:10.3389/fneur.2023.1148074)
Supplement: Supplementary file 3 [file Data_Sheet_1.PDF]

S1 Stroke Database Analysis

Novel analyses of the NINDS-tPA Stroke Trials Public Database and FAST-MAG Trial Core Database[1] was performed; both databases are publicly available by request from NIH-NINDS[2]. In the NINDS-tPA Stroke Trials analysis, participants in the placebo and treatment groups with an ischemic stroke, a pre-stroke mRS of 0 (no prior disability), and an mRS of 3 or 4 on day 7-10 were included in the analysis. In the FAST-MAG trials analysis, participants in the placebo and treatment groups with an ischemic stroke, a pre-stroke mRS of 0 (no prior disability), and an mRS of 3 or 4 on day 4 were included in the analysis. Datasets were analyzed for change in mRS scores from the subacute timepoint (day 7-10 in NINDS, day 4 in FAST-MAG) to 90 days. In both studies, participants improved by a mean of ~1 point at day 90 post-stroke. In both of these multicenter studies, patients received post-stroke rehabilitation care via many inpatient and outpatient rehabilitation facilities nationally and regionally. Patient outcomes may therefore be considered representative of those associated with the standard of care within the subacute to 90-day time period in the United States.

[1] Publicly available database of the NIH Field Administration of Stroke Therapy – Magnesium (FAST-MAG) Trial. Accessible to all upon request to the NIH-NINDS Archived Clinical Research Dataset repository. https://www.ninds.nih.gov/Current-Research/Research-Funded-NINDS/Clinical-Research/Archived-Clinical-Research-Datasets. Trial database finalized 2014. NINDS dataset website accessed Dec 2020.

[1] Archived Clinical Research Datasets" NIH makes them available to all requesters. The process for submitting requests for the datasets and listing of all available datasets is detailed at the following NIH URL: https://www.ninds.nih.gov/current-research/research-funded-ninds/clinical-research/archived-clinical-research-datasets

[1] Publicly available database of the NIH Field Administration of Stroke Therapy – Magnesium (FAST-MAG) Trial. Accessible to all upon request to the NIH-NINDS Archived Clinical Research Dataset repository. https://www.ninds.nih.gov/Current-Research/Research-Funded-NINDS/Clinical-Research/Archived-Clinical-Research-Datasets. Trial database finalized 2014. NINDS dataset website accessed Dec 2020.

[2] Archived Clinical Research Datasets" NIH makes them available to all requesters. The process for submitting requests for the datasets and listing of all available datasets is detailed at the following NIH URL: https://www.ninds.nih.gov/current-research/research-funded-ninds/clinical-research/archived-clinical-research-datasets
