# Supplementary material for: EMAGINE–Study protocol of a randomized controlled trial for determining the efficacy of a frequency tuned electromagnetic field treatment in facilitating recovery within the subacute phase following ischemic stroke
Source: Front Neurol. 2023 May 5;14:1148074. doi: 10.3389/fneur.2023.1148074 (PMC10196621; doi:10.3389/fneur.2023.1148074)
Supplement: Supplementary file 4 [file Data_Sheet_2.PDF]

S2 Ethics

This clinical trial will be conducted in accordance with: a) the Helsinki Declaration adopted by the 18th World Medical Assembly in Helsinki, Finland, in 1964, as last amended by the World Medical Assembly; b) the International Conference on Harmonisation of Technical Requirements for Registration of Pharmaceuticals for Human Use (ICH) Harmonised Tripartite Guideline for Good Clinical Practice (GCP) E6 (R2); c) the ISO 14155 standard, to the extent of recognition by the local regulations, and, where applicable; d) 21 CFR Parts 11, 50, 54, 56, 812; 45 CFR Parts 46 and 160; European Union (EU) Directive 93/42/EEC and, once applicable, Regulation 2017/745.

The protocol, informed consent form(s), recruitment materials, and all participant materials have been reviewed and approved by the central Institutional Review Board (IRB). Approval of both the protocol and the consent form was obtained before any participant was consented/enrolled. Any amendment to the protocol will require review and approval by the IRB before the changes are implemented in the study. All changes to the consent form will be approved by the IRB; a determination will be made regarding whether new consent needs to be obtained from participants who provided consent using a previously approved consent form.

The sponsor has taken out an insurance policy for the total duration of the study covering the participants and investigators with respect to the risks involved in conducting the study according to the protocol.

The investigator, or a person designated by the investigator, must fully inform the participant or, if the participant is unable to provide informed consent, the participant's legally acceptable representative, of all pertinent aspects of the trial including the written information given approval/favorable opinion by the IRB.
