# Supplementary material for: EMAGINE–Study protocol of a randomized controlled trial for determining the efficacy of a frequency tuned electromagnetic field treatment in facilitating recovery within the subacute phase following ischemic stroke
Source: Front Neurol. 2023 May 5;14:1148074. doi: 10.3389/fneur.2023.1148074 (PMC10196621; doi:10.3389/fneur.2023.1148074)
Supplement: Supplementary file 5 [file Data_Sheet_3.PDF]

S3 Recruitment Strategies

Strategies for achieving sufficient participant enrollment to reach the target sample size include: The sponsor's public websites or social media accounts may be used to advertise the trial, including a website dedicated to the EMAGINE trial (https://emagine.care/). Posters and/or flyers may also be issued to advertise the trial. Sites will have a dedicated tablet for presenting patients and families with information about the study that will include study-related videos and other recruitment materials. All recruitment materials will only be used following documented IRB approval. Additionally, the trial listing on ClinicalTrials.gov will be publicly accessible
