# Supplementary material for: EMAGINE–Study protocol of a randomized controlled trial for determining the efficacy of a frequency tuned electromagnetic field treatment in facilitating recovery within the subacute phase following ischemic stroke
Source: Front Neurol. 2023 May 5;14:1148074. doi: 10.3389/fneur.2023.1148074 (PMC10196621; doi:10.3389/fneur.2023.1148074)
Supplement: Supplementary file 6 [file Data_Sheet_4.pdf]

S4 Analysis Population and Missing Data

The following analysis sets are defined for this study:
Intent-to-treat (ITT) – will consist of all participants randomized. In accordance with the ITT principle, all participants randomized will be kept in their originally assigned treatment group.
Modified ITT (mITT) – will consist of all participants from the ITT analysis set for whom treatment was initiated and will be analyzed as randomized.
Per-protocol (PP) – includes all participants from the mITT analysis set who have no significant protocol deviations, were treated for a minimum of 20 completed sessions, and have at least one post-baseline data point; will be analyzed as treated.
Significant protocol deviations include, but are not limited to:

- enrollment in violation of the study eligibility criteria;
- performing the trial procedure in a manner inconsistent with that prescribed in the device instructions for use (IFU);
- other ethical or clinical considerations at the discretion of the investigator.
- enrollment in another interventional study overlapping with the period of participation in this study.

Safety (SAF) – will consist of all participants who initiated treatment, with participants analyzed as treated.
The SAF analysis set will serve as the principal analysis set for the analysis of safety.
The mITT analysis set will serve as the principal analysis set for efficacy assessments (including for secondary endpoints).
Efficacy assessments (including for secondary endpoints) will be performed on the PP as well as the ITT analysis set as a sensitivity analysis.
With regards to missing data – for the mRS up to 90 days post-stroke (primary endpoint), a high proportion of dropouts is not expected, as a placebo effect of the sham treatment is anticipated and was considered in the design of the study. Thus, any missing data within this time window is considered missing at random. Likelihood-based methods ensure valid inferences in this case.
A sensitivity analysis using methods for data imputation will be performed, specifically the last observed value (LOV) of that endpoint. The LOV is defined as the last available post-baseline visit data up to and including the last treatment visit or termination visit. Additionally, we may consider multiple imputation using a model incorporating key baseline prognostic features as well as the last post-randomization if deemed necessary.
