# Supplementary material for: EMAGINE–Study protocol of a randomized controlled trial for determining the efficacy of a frequency tuned electromagnetic field treatment in facilitating recovery within the subacute phase following ischemic stroke
Source: Front Neurol. 2023 May 5;14:1148074. doi: 10.3389/fneur.2023.1148074 (PMC10196621; doi:10.3389/fneur.2023.1148074)
Supplement: Supplementary file 7 [file Data_Sheet_5.pdf]

S5 Discontinuation

Participants may withdraw from the study or discontinue the study intervention at any time. Participation may be terminated by the investigator or sponsor due to an unexpected serious AE, pregnancy, or a significant protocol deviation. Data collected prior to withdrawal of consent will remain in the study database, but no further data will be collected.
