# Supplementary material for: EMAGINE–Study protocol of a randomized controlled trial for determining the efficacy of a frequency tuned electromagnetic field treatment in facilitating recovery within the subacute phase following ischemic stroke
Source: Front Neurol. 2023 May 5;14:1148074. doi: 10.3389/fneur.2023.1148074 (PMC10196621; doi:10.3389/fneur.2023.1148074)
Supplement: Supplementary file 8 [file Data_Sheet_6.pdf]

S6 EMAGINE Investigators and coordinators

Atrium Health Carolinas Rehabilitation (The Charlotte-Mecklenburg Hospital Authority)
Dr. Matthew Shall (PI), Tami Pringnitz Guerrier, Christine Patino, Brian Adams
Baylor Scott & White Institute for Rehabilitation
Dr. Simon Driver (Co-PI), Dr. Rosemary Dubiel (Co-PI), Claudia Cohen, Lacy McDonald
Brooks Rehabilitation Hospital – University Campus
Dr. Emily Fox (PI), Dr. Lou DeMark, Dr. Cassandra List, Dr. Meghan Kettles, Hannah Snyder
Christine E. Lynn Rehabilitation Center for The Miami Project/Jackson Memorial Hospital
Dr. Sebastian Koch (PI), Dr. Lauren Shapiro (Co-PI), Dr. W. Dalton Dietrich, III, Iszet Campo-Bustillo, Dr. Nilanjana Datta
Kessler Institute of Rehabilitation
Dr. Steven Kirshblum (PI), Dr. Ghaith Androwis (Co-PI), Dr. Guang Yue, Dr. Irene Ward, Dr. Amanda Engler, Sally Al-Rabadi
MedStar National Rehabilitation Hospital (NRH)
Dr. Richard Zorowitz (PI), Kathaleen Brady
Moss Rehabilitation Research Institute (MRRI)
Dr. Ning Cao (PI), Dr. Alberto Esquenazi, Dr. Dylan Edwards, Jaun May, Grace Loscalzo, Tori Sprowl
NYP Brooklyn Methodist Hospital Outpatient Rehabilitation
Dr. Joel Stein (PI), Dr. Jason Edwards (Co-PI), Dr. Holly Batistick-Aufox, Joy Rutherford
Shirley Ryan Abilitylab
Dr. Elliot Roth (PI), Dr. Arun Jayaraman (Co-PI), Dr. Sara Prokup, Dr. Joe Harris
Spaulding Rehabilitation Hospital
Dr. Randie Black-Schaffer (PI), Dr. David Lin (Co-PI), Dr. Paolo Bonato, Dr. Priyanca Shah, Jenny Min
TIRR Memorial Hermann Hospital
Dr. Sean Savitz (PI), Emily Stevens
The University of Kansas Medical Center (KUMC)
Dr. Sandra Billinger (Co-PI), Dr. Sarah Eickmeyer (Co-PI), Bria Bartsch, Sasha (Alexandra) Moores
UCLA and California Rehabilitation Institute
Dr. Mersedeh Bahr Hosseini (Co-PI), Dr. Michael Su (Co-PI:), Dr. Jeffery Saver, Dr. Steven Cramer, Dr. Pamela Roberts, Rocky Pang, Dr. Adrienne Ingalla, Gilda Avila
BrainQ Technologies Ltd.
Assaf Lifshitz, Arielle Hochberg, Yotam Drechsler, Yael Djemal Kay, Batsheva Weisinger, Dr. Yaron Segal, Dr. Nadav Bitton, Iren Basanov, Dr. Allison Nogi, Dr. Vincent Santucci, Dr. Jessica Royal, Dr. Ana Parabucki, Dr. Adina Bitton, Prof. Esther Shohami
